# Long non-coding RNA NCK1-AS1 promotes the tumorigenesis of glioma through sponging microRNA-138-2-3p and activating the TRIM24/Wnt/β-catenin axis

**DOI:** 10.1186/s13046-020-01567-1

**Published:** 2020-04-15

**Authors:** Lifa Huang, Xu Li, Hui Ye, Yajun Liu, Xiaolong Liang, Chao Yang, Lin Hua, Zhaoxian Yan, Xin Zhang

**Affiliations:** 1 Department of Neurosurgery, Zhejiang Provincial Hospital of Traditional Chinese Medicine/The First Affiliated Hospital of Zhejiang Chinese Medical University, No. 54, Youdian Road, Shangcheng District, Hangzhou, Zhejiang 310006 People’s Republic of China; 2 The First Clinical Medical College, Zhejiang Chinese Medical University, Hangzhou, Zhejiang 310053 People’s Republic of China

**Keywords:** Long non-coding RNA NCK1-AS1, microRNA-138-2-3p, TRIM24, Wnt/β-catenin signaling pathway, Glioma

## Abstract

**Background:**

Glioma is a common brain malignancy with high mortality. The competing endogenous RNA (ceRNA) networks may play key roles in cancer progression. This study was conducted to probe the role of long noncoding RNA (lncRNA) NCK1-AS1 in glioma progression and the involved mechanisms.

**Methods:**

Microarray analyses were performed to explore the lncRNAs/miRNAs/genes with differential expression in glioma. NCK1-AS1 levels in glioma tissues and normal brain tissues, and in glioma cell lines and normal human glial cells were identified. The interactions among NCK1-AS1, miR-138-2-3p and TRIM24 were validated through luciferase reporter, RNA immunoprecipitation and RNA pull-down assays. Gain- and loss-of functions of NCK1-AS1, miR-138-2-3p and TRIM24 were performed to identify their roles in the behaviors of glioma cells. The activity of the Wnt/β-catenin pathway was measured. In vivo experiments were performed as well.

**Results:**

High expression of NCK1-AS1 was found in glioma tissues and cells, especially in U251 cells. Online predictions and the integrated experiments identified that NCK1-AS1 elevated the TRIM24 expression through sponging miR-138-2-3p, and further activated the Wnt/β-catenin pathway. Artificial silencing of NCK1-AS1 or up-regulation of miR-138-2-3p led to inhibited proliferation, invasion and migration but promoted cell apoptosis of U251 cells, while up-regulation of TRIM24 reversed these changes, and it activated the Wnt/β-catenin pathway. The in vitro results were reproduced in in vivo experiments.

**Conclusions:**

Our study suggested that NCK1-AS1 might elevate TRIM24 expression and further activate the Wnt/β-catenin pathway via acting as a ceRNA for miR-138-2-3p. Silencing of NCK1-AS1 might inhibit the progression of glioma.

## Background

Gliomas are tumors that initiate from precursor or glial cells and include astrocytoma (including glioblastoma), oligodendroglioma, ependymoma, oligoastrocytoma (mixed glioma) and malignant glioma and account for about 25.5% of all primary brain and other central nervous system tumors and 80.8% of malignant tumors [1]. The current clinical treatment of glioma includes surgery, chemotherapy, radiotherapy, targeted therapy and immunotherapy; however, the prognosis of glioma remains unfavorable with a high recurrence rate after initial treatment [2]. Diffuse glioma comprises no more than 1% of all newly diagnosed cancers, though, it leads to high mortality and morbidity, especially the most lethal type, glioblastoma, which takes place with 70–75% of all glioma cases with a median overall survival of 14–17 months [3]. Developing novel therapeutic options and identifying new molecular mechanisms are of great importance in glioma control.

Long non-coding RNAs (lncRNAs) and microRNAs (miRNAs) are two large classes of non-protein-coding transcripts that participate in diverse essential cellular processes through multiple mechanisms [4, 5]. LncRNAs are larger than 200 nucleotides, and their importance in gene expression, key cellular processes, metastasis and disease prognosis in cancer has been largely studied [6, 7], including in glioma [8, 9]. miRNAs are 20–25 nucleotides in length, and they down-regulate gene expression by binding with target mRNAs post-transcriptionally thus resulting in transcript degradation [10]. miRNAs exert critical functions in fundamental cellular process such as cell proliferation, apoptosis, development and inflammation [11]. Likewise, dysregulation of several miRNAs has been found to participate in human glioma progression [12, 13]. Importantly, a competing endogenous RNA (ceRNA) theory proposed recently, which suggests that non-coding RNAs and protein-coding RNAs work as ceRNAs through competing for miRNAs via shared miRNA recognition elements [14, 15], has aroused wide concerns. Several ceRNA networks have been validated to play key functions in metastasis, proliferation, and apoptosis in human glioma cells [16, 17]. The possible ceRNA networks in glioma and the mechanisms involved remain largely unknown.

To this end, this study first figured out the representative differentially expressed lncRNAs through the glioma microarrays, and lncRNA NCK1-AS1, which has been noted to be aberrantly expressed in several human cancers [18, 19] with its role in glioma remaining unknown, was selected as the subject of this study. We further determined tripartite motif-containing 24 (TRIM24) as an indirect target of NCK1-AS1 through the miR-138-2-3p sponge. TRIM24 is a member of the TRIM family with its oncogenic role being identified in prostate cancer [20]. In addition, TRIM24 has been suggested to promote cancer progression by triggering the Wnt/β-catenin signaling pathway [21, 22]. This pathway has been noted to be closely associated with cellular proliferation, migration, and angiogenesis and the following glioma malignancy [23]. We assumed that NCK1-AS1 could affect glioma progression through the miR-138-2-3p/TRIM24 network and the following Wnt/β-catenin pathway, with gain- and loss-of functions of these molecules performed in both cell and animal experiments to validate this hypothesis.

## Materials and methods

### Microarray analysis

The glioma gene microarrays (GSE50161 and GSE35493) and miRNA microarray (GSE65626) acquired from the GEO Database (https://www.ncbi.nlm.nih.gov/geo/) were used for differential expression analysis utilizing an R-Language Limma Package [24]. Differentially expressed lncRNAs/miRNAs/genes were analyzed with |log_2_FC| > 1.0 and *p* < 0.05 as the criteria, and the heatmaps for differentially expressed lncRNAs/miRNAs/genes were drawn by an online pheatmap package (https://cran.r-project.org/web/packages/pheatmap/index.html). The binding sites between lncRNAs and miRNAs were predicted on the RNA22 database (https://cm.jefferson.edu/rna22/). Besides, the RNA22, miRDB (http://www.mirdb.org), miRDIP (http://ophid.utoronto.ca/mirDIP/), miRWalk (http://mirwalk.umm.uni-heidelberg.de/) and DIANA (http://diana.imis.athena-innovation.gr/DianaTools/index.php?r=microT_CDS/index) databases were applied to predict the target genes of miRNAs. The prediction outcomes were integrated and analyzed using a Venn diagram produced by online webtools (http://bioinformatics.psb.ugent.be/webtools/Venn/).

### Clinical tissue sample collection

A total of 12 pairs of normal brain tissue samples and 32 pairs of glioma tissue samples were acquired from the Zhejiang Provincial Hospital of Traditional Chinese Medicine. Normal brain tissues collected from epileptic patients who subjected to surgery were used as control. The study gained the approval of the Clinical Ethical Committee of the Zhejiang Provincial Hospital of Traditional Chinese Medicine. All procedures were conducted as per the *Declaration of Helsinki.* All eligible participants signed the informed consent.

### RNA-in situ hybridization (RNA-ISH)

RNA-ISH of lncRNA NCK1-AS1 was performed using an ISH assay kit (Boye Biotechnology Co., Ltd., Guangzhou, Guangdong, China). In brief, the tissue samples were fixed, embedded in paraffin, and warm-incubated in gradient alcohol and then in 3% H_2_O_2_ for 30 min. Then, the streptavidin-horseradish peroxidase (HRP) conjugate and biotin conjugate probes were introduced into the samples for hybridization. Then samples were then stained with hematoxylin and observed under an optical microscope (Leica, Solms, Germany).

### Cell culture

Normal human glial cell line HEB from Yan-Yu Bio-technology Co., Ltd. (Shanghai, China) (http://www.hdbsw.com/) and 4 glioma cell lines U251, SHG-441, U87 and T98 (ATCC, Manassas, USA) were incubated in DMEM (Gibco, NY, USA) supplemented with 10% fetal bovine serum (FBS), penicillin (100 U/mL) and streptomycin (100 mg/mL) (37 °C with 5% CO_2_). The cells were passaged when they reached an 85% confluence.

### Cell treatment and grouping

The glioma cells were trypsinized to 2 × 10^6^ cells/mL. Then the cell suspension was sorted into 12-well plates at 1 mL per well and incubated in 5% CO_2_ at 37 °C. Next, the cells were transfected with different vectors using the Lipofectamine 2000 (Thermo Fisher, MA, USA) as per the protocols and allocated into the corresponding groups. Three batches of grouping were performed. As for the first batch, cells were allocated into over-expression (oe)-negative control (NC) group (oe-NC group, cells were transfected with NC of NCK1-AS1 oe vector), oe-NCK1-AS1 group (cells were transfected with NCK1-AS1 oe vector), short hairpin (sh)-NC group (cells were transfected with NC of shRNA of NCK1-AS1) and sh-NCK1–AS1 (cells were transfected with sh-NCK1-AS1).

As for the second batch, cells were allocated into mimic NC group (cells were transfected with mimic NC), miR-138-2-3p mimic group (cells were transfected with miR-138-2-3p mimic), inhibitor NC group (cells were transfected with inhibitor NC) and miR-138-2-3p inhibitor group (cells were transfected with miR-138-2-3p inhibitor).

In terms of the third batch, cells were allocated into mimic NC group, miR-138-2-3p mimic group, inhibitor NC group, miR-138-2-3p inhibitor group, miR-138-2-3p mimic + oe-NC group (cells were transfected with miR-138-2-3p mimic and NC of NCK1-AS1 oe vector) and miR-138-2-3p mimic + oe-TRIM24 group (cells were transfected with miR-138-2-3p mimic and TRIM24 oe vector).

After transfection, the cells were incubated in the transfection solution for 4 h and then in normal cell culture medium for following experiments. The sequences for the vectors or mimic are listed in Supplementary Table 1.

### Dual luciferase reporter gene assay

The wide type (WT) sequence based on the binding site between NCK1-AS1 and miR-138-2-3p and the corresponding mutant type (MUT) sequence were inserted into the target sequence of the psiCheck2 vector to construct psiChech2-NCK1-AS1-WT vector and psiChech2-NCK1-AS1-MUT vectors. The psiChech2-TRIM24-WT psiChech2-TRIM24-MUT vectors were constructed in a similar manner. Next, well-constructed reporter vectors were co-transfected with miR-138-2-3p mimic or mimic NC into HEK293T cells, and then the luciferase activity was determined in accordance with the instructions of a dual luciferase reporter assay system (Promega, Madison, WI, USA). Three independent experiments were performed.

### 5-ethynyl-2′-deoxyuridine (EdU) labeling assay

Exponentially growing cells were sorted into 24-well plates, and to each group, 3 duplicated cells were set up. Next, EdU (RiboBio Co., Ltd., Guangdong, China) was filled into the culture medium and adjusted to 10 μmol/mL. After 2 h of incubation in 5% CO_2_ at 37 °C_,_ the medium was absorbed, and the cells were fixed in 4% paraformaldehyde-supplemented phosphate buffer saline (PBS) for 15 min, washed twice in 3% bovine serum albumin (BSA)-supplemented PBS, and then incubated in PBS supplemented with 0.5% Triton-100 for 20 min. Following two 3%BSA-PBS washes, each well was filled with 100 μL Apollo® 567 (RiboBio) and incubated for 30 min without light exposure at room temperature. Then the cells were washed twice in 3% BSA-PBS again, and stained with 1× Hoechst 33342 for 30 min. Thereafter, the wells were washed 3 more times in PBS, sealed, and observed under a fluorescence microscope (Olympus Optical Co., Ltd., Tokyo, Japan) with 5 views randomly selected. The positive cells, which were stained in red under the scope, were counted and recorded. The procedures were repeated 3 times.

### Scratch test

Forty-eight hours after transfection, each group of cells were sorted into 6-well plates at 5 × 10^5^ cells/well. A 200 μL pipette tip (21–0200, Biologix, Shanghai, China) was used to produce a scratch through the midline of the wells when the confluence reached 85%. Then the floating cells were washed away with PBS, while the remaining cells were continually incubated in serum-free medium for 1 h of recovery. At 0 h and 24 h after recovery, the cells were photographed to measure the migration of cells on an Image-Pro Plus Anaysis software (Media Cybernetics, USA). The experiment was performed in triplicate.

### Transwell assay

Extracellular matrix (ECM) gel was allowed to stand at 4 °C overnight. The next day (all the pipette tips and Transwells were pre-cooled on ice for half an hour), the gel was diluted in serum-free medium at 1:9 till the final concentration reaching 1 mg/mL. Each apical chamber of the 24-well Transwells was loaded with 40 μL ECM gel on the polycarbonate membrane and then placed in a 37 °C incubator with 5% CO_2_ for 5 h to polymerize the ECM gel. Next, the remaining liquid was removed, and each well was filled with 70 μL DMEM and incubated at 37 °C with 5% CO_2_ for 30 min to hydrate the gel again with the remaining culture medium discarded. After that, each group of cells were hungered in a serum-free condition for 24 h, detached, centrifuged, and diluted in FBS-free DMEM to 2.5 × 10^5^ cells/mL. Then 0.2 mL suspension was loaded in each apical chamber where the substrate membraned was already hydrated, while each basolateral chamber was loaded with 700 μL pre-cooled 10% FBS-DMEM. The chambers were then incubated in 5% CO_2_ with saturated humidity at 37 °C for 24 h. Thereafter, the apical chambers were removed, and the cells in apical chambers and on the substrates were discarded using wet cotton swabs. The remaining cells were methanol-fixed, stained by 0.1% crystal violet, air dried, and photographed under a microscope with 5 random fields (200 ×) observed, and the average volume of invaded cells was calculated. The experiments were performed in triplicate.

### Flow cytometry

Cells were washed 3 times in PBS 48 h after transfection and centrifuged at 3000 r/min for 20 min to discard the supernatant, and then diluted in PBS to adjust the concentration to 1 × 10^5^ cells/mL. Then the suspension was successively treated with 1 mL − 20 °C pre-cooled 75% ethanol for 1 h of fixing, centrifuged for 5 min at 1500 r/min, washed in PBS, treated with 100 μL Rnase A (Thermo Fisher) without light exposure, bathed in 37 °C water for 30 min, and stained by 400 μL propidium iodide (PI) (Sigma-Aldrich, Merck KGaA, Darmstadt, Germany) at 4 °C for 30 min. Next, the cell cycle was measured based on the red fluorescence at the 488 nm (excitation wavelength).

In terms of apoptosis detection, the cells were detached in EDTA-free trypsin (Thermo Fisher) 48 h after transfection and filled into the flow cytometer tubes. Following 30 min of centrifugation at 3000 r/min and 3 cold PBS washes, the cells were further centrifuged at 3000 r/min for 20 min to discard the supernatant. Then the Annexin-V-FITC/PI staining solution was compounded by HEPES buffer (Thermo Fisher), Annexin-V-FITC and PI (50:1:2). Then the cells were stained for 15 min at room temperature, and then treated with 1 mL HEPES buffer. After vibration, the cell apoptosis was measured on the flow cytometer at 488 nm.

### Fluorescence in situ hybridization (FISH)

The sub-cellular localization of NCK1-AS1 in glioma cell line was assessed according to the protocols of a lncRNA FISH probe Mix (RiboBio). In brief, cover glasses were put into 6-well plates on which glioma cells were seeded. One day later when the cell confluence got to 80%, the cell slides were collected, washed in PBS, and treated with 1 mL 4% paraformaldehyde. Next, the cells were given Protease-K (2 μg/mL), glycine and acetylating agent, and then cultured with prehybridization agent (250 μL) at 42 °C for 1 h, and then with hybridization agent (250 μL) containing probes (300 ng/mL) at 42 °C overnight. After 3 PBST washes, the cells were treated with PBST-DAPI (1:800) for nucleus staining for 5 min. Next, the cell slides were washed in PBST (3 × 3 min), sealed with anti-fluorescence quencher, and then observed under the fluorescence microscope (× 400) with 5 random fields included.

### RNA immunoprecipitation (RIP)

A RIP kit (Millipore Corp., Billerica, MA, USA) was applied to measure the binding relation between NCK1-AS1 and AGO2. Glioma cells were washed in PBS. Then the cells were lysed in an equal volume of RIPA cell lysis solution (Beyotime, Shanghai, China) on ice for 5 min, and then centrifuged at 14000 r/min at 4 °C to collect the supernatant. A part of cell lysis was used as Input, and another part was cultured with the antibodies for coprecipitation. In each coprecipitation system, 50 μL magnetic beads were resuspended in RIP wash buffer (100 μL) and then incubated with 5 μg antibodies according the grouping. The magnet bead-antibody compounds were resuspended in RIP wash buffer (900 μL), and further cultured with 100 μL cell lysis overnight at 4 °C. The samples were set on the magnet base to obtain the magnet bead-antibody compounds. The samples and Input were respectively detached with protease K with the RNA extracted for the following reverse transcription quantitative polymerase chain reaction (RT-qPCR). The antibodies are AGO2 (ab32381, 1:50) and immunoglobulin G (IgG, 1:100, A109489) (all provided by Abcam Inc., Cambridge, MA, USA).

### RNA pull-down

Glioma cells were either transfected with MUT-biotinylated or WT-biotinylated miR-138-2-3p (50 nM for each). Cells were harvested 48 h after, washed in PBS, and whirled. Then the cells were cultured for 10 min in specific cell lysis solution (Ambion, Austin, Texas, USA). The cell lysates were co-cultured with RNase-free and yeast tRNA-precoated M-280 streptavidin beads (all from Sigma-Aldrich) for 3 h at 4 °C and then washed twice in cold lysis solution, 3 times in low salt buffer, and once in high salt buffer. The combined RNA was purified with Trizol, and the NCK1-AS1 expression was evaluated using RT-qPCR.

### Western blot analysis

Glioma cells and tissues were lysed in protease inhibitor-supplemented enhanced RIPA lysis solution (Boster, Wuhan, China) and then to have the total protein collected, and a bicinchoninic acid kit (Boster) was applied to assess the protein concentration. The proteins were run on 10% SDS-PAGE and transferred onto PVDF membranes. Then the membranes were sealed in 5% BSA for 2 h to block non-specific binding, and then cultured with the primary antibodies (Table 1) at 4 °C overnight. Afterwards, the membranes were washed 3 times in PBST and incubated with horseradish peroxidase-labeled goat-anti-rabbit (ab205719, 1:2000, Abcam) for 1 h at room temperature. Then the membranes were further washed in PBST and measured using the enhanced chemiluminescence reagent (EMD Millipore, USA). The signal intensity of the protein bands was analyzed using Image J software with the value of GAPDH set as the internal reference. The procedures were conducted for 3 times.

**Table 1:** Antibodies used in western blot analysis

Antibodies	Item. No.	Dilution ratio
PCNA	ab18197	1:1000
N-cadherin	ab76011	1:5000
MMP-9	ab38898	1:1000
Bcl-2	ab196495	1:1000
Bax	ab53154	1:1000
Wnt1	ab15251	1:5000
Wnt3a	ab28472	1:2000
β-catenin	ab6302	1:4000
GAPDH	ab37168	1:2000

Note: All antibodies were purchased from Abcam Inc., Cambridge, MA, USA *PCNA* proliferating cell nuclear antigen, *MMP-9* matrix metalloproteinase-9, *Bcl-2* B-cell lymphoma-2, *Bax* Bcl-2-associated X, *GAPDH* glyceraldehyde-3-phosphate dehydrogenase

### RT-qPCR

The Trizol reagent (15,596,026, Invitrogen Inc., Carlsbad, CA, USA) was utilized to obtain the total RNA of cells and tissues. Reverse transcription was performed using a PrimeScript RT reagent kit (RR047A, Takara Holdings Inc., Tokyo, Japan) to produce cDNA. Quantification was performed as per the instructions of a RT-qPCR assay kit (Thermo Fisher). The relative expression of miR-148a was normalized by a U6 transcript. Three duplicate wells were set for each group. U6 was set as the internal reference for miR-138-2-3p while GAPDH for other genes. Relative gene expression was evaluated using the 2^-ΔΔCt^ method. The primers were synthetized by TransGen Biotech (Beijing, China) and the sequences are presented in Table 2.

**Table 2:** Primer sequences for RT-qPCR

Gene	Primer sequence
NCK1-AS1	F: 5′-GCCGCAGGAGAGACTTAACA-3′
	R: 5′-CCTTCGGCTGGGATGACATT-3′
miR-138-2-3p	F: 5′-GCGACCCGATTGTCCATAAG-3′
	R: 5′-AGCAAGCCTGTCTGATGTGA-3′
TRIM24	F: 5′-CATATGCAGCAACAGCAACCG-3′
	R: 5′-GAAAGCCATCTGTAGGGGGT-3′
β-catenin	F: 5′-CATATGCAGCAACAGCAACCG-3’
	R: 5′-GAAAGCCATCTGTAGGGGGT-3’
U6	F: 5′-CTCGCTTCGGCAGCACA-3’
	R: 5′-AACGCTTCACGAATTTGCGT-3’
GAPDH	F: 5′-GAAGACGGGCGGAGAGAAAC-3’
	R: 5′-CCATGGTGTCTGAGCGATGT-3’

Note: *RT-qPCR* reverse transcription quantitative polymerase chain reaction, *miR* microRNA, *TRIM24* tripartite motif containing 24, *GAPDH* glyceraldehyde-3-phosphate dehydrogenase

### Immunofluorescence staining

The expression of stemness-related proteins SOX2 (1:1000, ab97959, Abcam) and OCT4 (1:1000, ab18976, Abcam) in U251 and SHG441 cells was determined as guided by a previous study [25].

### Tumor sphere formation assay

U251 and SHG-441 cells were seeded into 6-well ultra-low attachment culture dishes (Corning Incorporated, Corning, NU, USA). Then the cells were cultured in serum-free DMEM/F12 supplemented with 1% B27, 20 ng/mL human epidermal growth factor (EGF) and 20 ng/mL human fibroblast growth factor (FGF) at 37 °C for 2 weeks to form tumor spheres. Two weeks later, the number of newly formed tumor spheres were counted under an optical microscope (Nikon Corporation, Tokyo, Japan) at a × 200 magnification.

### Xenograft tumors in nude mice

Thirty female BALB/c nude mice (3–4 weeks old, 14 ± 2 g, provided by SJA Laboratory Animal Co., Ltd., Hunan, China) were fed at 25–27 °C with controlled humidity (45–50%) with free access to food and water. Then the mice were randomized into 6 groups: sh-NC group, sh-NCK1-AS1 group, mimic NC group, miR-138-2-3p mimic group, sh-NCK1-AS1 + oe-NC group and sh-NCK1-AS1 + oe-TRIM24 group, 5 mice in each. Cells with stable transfection were resuspended and adjusted to 1 × 10^7^ cells/mL, and each nude mouse was implanted with 20 μL cell suspension. The tumor growth was observed and photographed every 7 d for a total of 35 d, and a tumor growth curve was drawn. The tumor volume was calculated as (a × b^2^)/2, in which “a” refers to the longest diameter while “b” refers to the shortest. The mice were euthanized on the 35th d via intraperitoneal injection of 150 mg/kg pentobarbital to collect and weigh the tumors. Thereafter, the tumor samples were preserved in liquid nitrogen for following RT-qPCR and western blot analysis. Animal studies were conducted as per the principles and procedures ratified by the Committee on the Ethics of Animal Experiments of the Zhejiang Provincial Hospital of Traditional Chinese Medicine. Great attempts were made to minimize the number and pain of animals.

### Statistical analysis

SPSS 21.0 (IBM Corp. Armonk, NY, USA) was applied for data analysis. Data were in normal distribution. Measurement data were exhibited as mean ± standard derivation (mean ± SD). Differences between every two groups were analyzed using the *t* test, while those among multiple groups were analyzed using one-way analysis of variance (ANOVA) and Tukey’s multiple comparison test. Differences among multiple groups at different time points were analyzed using repeated measurement ANOVA and Bonferroni test. The Log-rank test was used for single-factor analysis. The *p* value was acquired from two-tailed tests, and *p* < 0.05 was considered to show significant difference.

## Results

### NCK1-AS1 is highly expressed in PC tissues

**Fig. 1:**
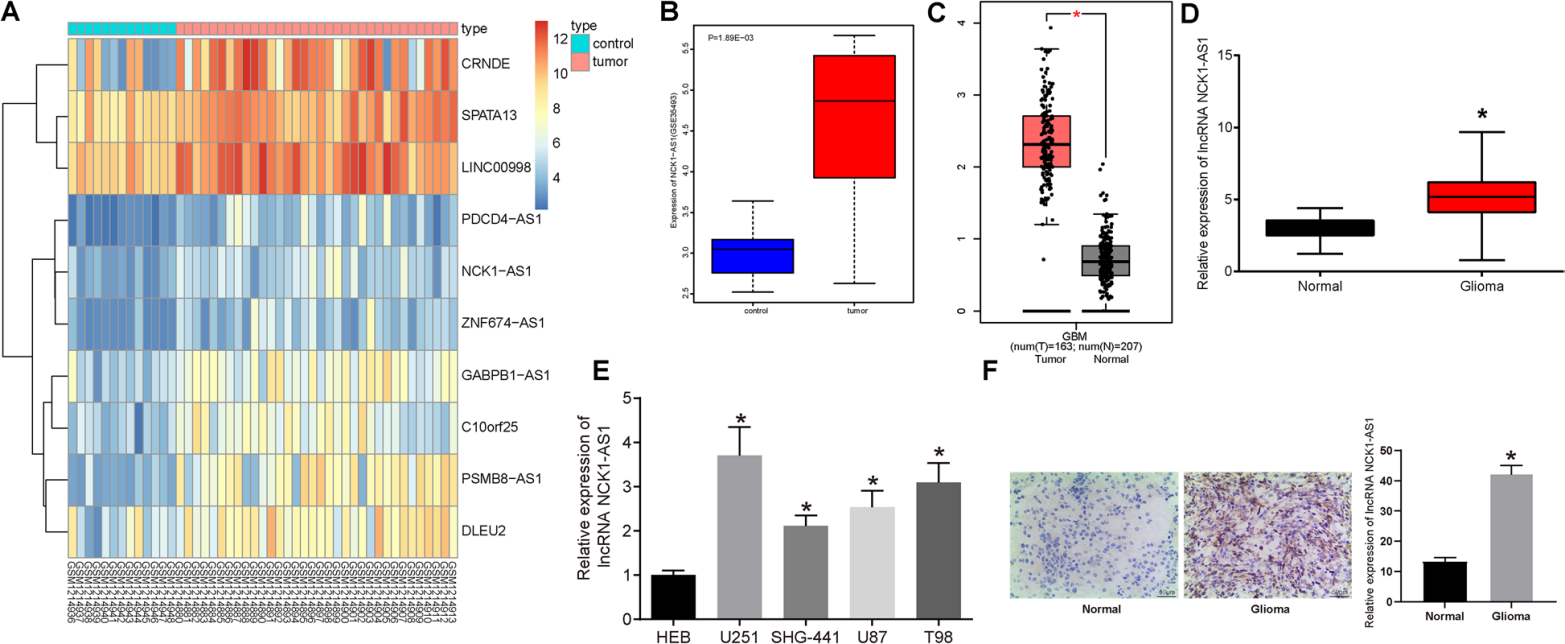
NCK1-AS1 is highly expressed in PC tissues. **a** heatmap for top 10 highly-expressed lncRNAs in the glioma microarray GSE50161, in which the abscissa refers to the sample numbers while the ordinate refers to the differentially expressed lncRNAs; the histogram in upper right corner is the color scale, in which each rectangle indicates the expression for each sample; **b** NCK1-AS1 expression in glioma microarray GSE35493; **c** NCK1-AS1 expression in TCGA-GBM predicted on GEPIA (http://gepia.cancer-pku.cn/) (T, tumor; N, normal); **d** NCK1-AS1 expression in glioma and normal brain tissue samples measured using RT-qPCR, * compared to the Normal tissues, *p* < 0.05; E, NCK1-AS1 expression in glioma cell lines U251, SHG-441, U87 and T98 and in human glial cell line HEB measured using RT-qPCR, * compared to the HEB cells, *p* < 0.05; F, NCK1-AS1 expression in brain tissues and glioma tissues validated using RNA-ISH assay, * compared to the HEB cells, *p* < 0.05. Measurement data were measured using mean ± SD; difference between two groups was measured using the unpaired *t* test, while differences among multiple groups were analyzed using one-way ANOVA

Differentially expressed lncRNAs in the glioma microarrays GSE50161 and GSE35493 were analyzed, and the heatmap for top 10 highly expressed lncRNAs in the microarray GSE50161 was produced (Fig. 1a). And we chose NCK1-AS1, whose oncogenic roles have been noted in many other cancer types excluding glioma, as the subject of this study. Besides, high expression of NCK1-AS1 was also found in the other microarray GSE35493 (Fig. 1b). The online system GEPIA (http://gepia.cancer-pku.cn/) suggested that NCK1-AS1 is highly expressed in the TCGA-GBM DataBase (Fig. 1c). Moreover, in line with the prediction results, RT-qPCR identified high NCK1-AS1 expression in glioma tissues (*p* < 0.05) (Fig. 1d). Still, the NCK1-AS1 expression was increased in glioma cell lines U251, SHG-441, U87 and T98 compared to the normal glial cell line HEB, with the highest expression found in the U251 cell line while the lowest in the SHG-441 cell line (all *p* < 0.05) (Fig. 1e). Then, the U251 and SHG-441 cell lines were used for the subsequent experiments. In addition, RNA-ISH was further performed to validate NCK1-AS1 expression in brain and glioma tissues, and the results also suggested a higher NCK1-AS1 level in glioma tissues than in brain tissues (Fig. 1f).

**Fig. 2:**
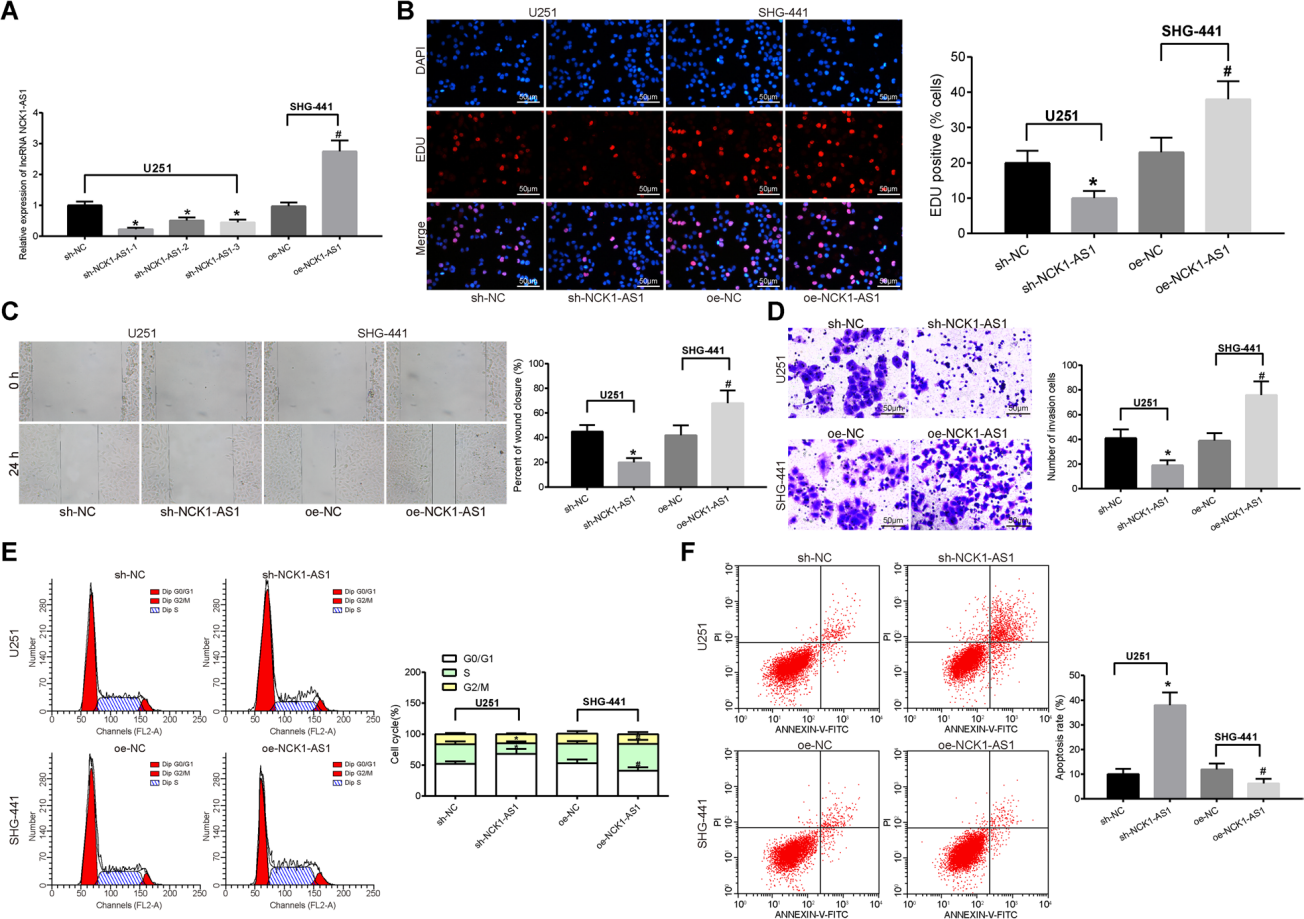
Silencing of NCK1-AS1 inhibits the malignant behaviors of U251 cells. **a**. NCK1-AS1 expression in U251 cells following interventions measured using RT-qPCR; **b** proliferation of U251 cells detected using EdU assay; **c** migration of U251 cells detected using the scratch test and observed under the microscope (× 100); **d** number of invaded cells in the Transwell assay observed under the microscope (× 100); E-F, cell cycle (**e**) and the apoptosis rate (**f**) of U251 cells measured using flow cytometry. * compared to the sh-NC group, *p* < 0.05; ^#^ compared to the oe-NC group, *p* < 0.05. Measurement data were measured using mean ± SD; differences among multiple groups were analyzed using one-way ANOVA; Repetition = 3

Artificial silencing of NCK1-AS1 was introduced in U251 cells while over-expression of NCK1-AS1 was introduced in SHG-441 cells. The transfection was successfully performed based on the NCK1-AS1 expression in cells measured by RT-qPCR, and the sh-NCK1-AS1–1 presented the best silencing efficacy (all *p* < 0.05) (Fig. 2a). The EdU assasy, scratch test and Transwell assay results suggested that silencing of NCK1-AS1 led to inhibited proliferation, migration and invasion of U251 cells, while over-expression of NCK1-AS1 resulted in opposite trends in SHG-441 cells (all *p* < 0.05) (Fig. 2b-d). Moreover, the flow cytometry results suggested that silencing of NCK1-AS1 led to increased cell ratio in the G0/G1 phase while decreased cells in the S phase, correspondingly the cell apoptosis rate was increased in U251 cells. Conversely, the cell cycle arrest in G0/G1 phase and the apoptosis of SHG-441 cells with over-expressed NCK1-AS1 were decreased (all *p* < 0.05) (Fig. 2e-f). These results indicated that silencing of NCK1-AS1 might inhibit proliferation, invasion, migration and the resistance to death of glioma cells.

### NCK1-AS1 might bind to miR-138-2-3p to regulate TRIM24

**Fig. 3:**
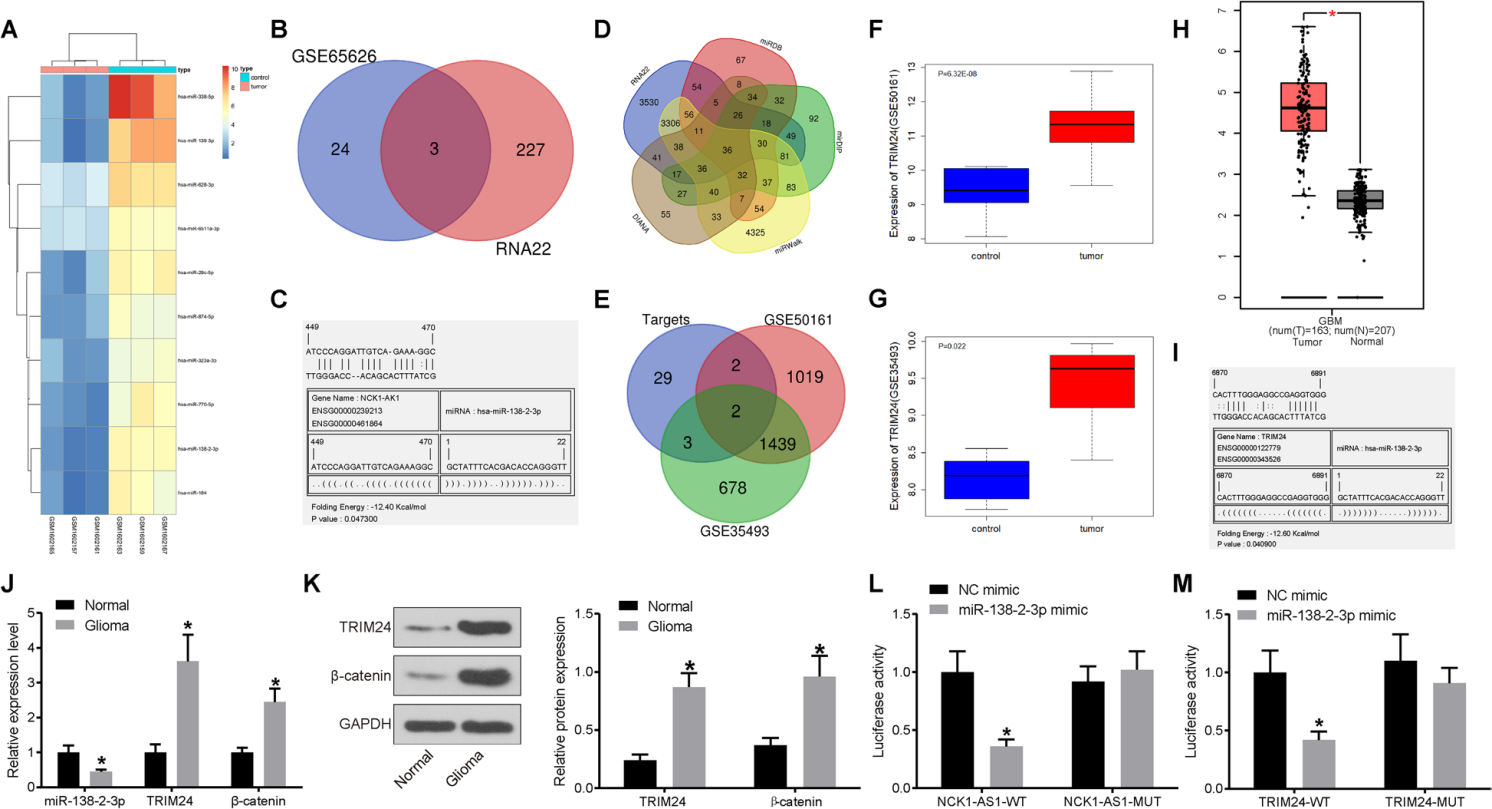
Potential molecular mechanisms in glioma predicted by bio-information systems. **a** a heatmap for top 10 differentially expressed miRNAs in glioma according to the data on GSE65626 microarray, in which the abscissa indicates sample number and the ordinate indicates miRNAs; the histogram in right top is the color scale, and each rectangle corresponds to a sample expression value (has, *Homo sapiens*); **b** a Venn diagram of the intersections between the downstream miRNAs of NCK1-AS1 predicted on RNA22 (https://cm.jefferson.edu/rna22/) and the lowly-expressed miRNAs in microarray GSE62656; **c** binding sites between NCK1-AS and has-miR-138-2-3p predicted on RNA22; **d** intersection of the target genes of miR-138-2-3p predicted on several online systems (RNA22, miRDB, mirDIP, miRWalk and DIANA); **e** above intersect genes compared with the highly expressed genes in the glioma microarrays GSE50161 and GSE35493; **f-g** TRIM24 expression in GSE50161 and GSE35493; **h** TRIM24 expression in the TCGA-GBM database (T: tumor; N, normal); **i**, binding sites between miR-138-2-3p and TRIM24 predicted on RNA22; **j-k**, expression of miR-138-2-3p, and mRNA expression of TRIM24 and β-catenin in glioma and normal brain tissue samples using RT-qPCR (**j**) and protein expression of TRIM24 and β-catenin by western blot analysis, respectively, * compared to the normal group, *p* < 0.05; L-M, binding relation between NCK1-AS1 and miR-138-2-3p, and between miR-138-2-3p and TRIM24 detected using dual luciferase reporter gene assay. * compared to the mimnic NC group, *p* < 0.05. Measurement data were measured using mean ± SD; differences between two groups were analyzed using the unpaired *t* test, while that among multiple groups were analyzed using one-way ANOVA; Repetition = 3

A heatmap for top 10 differentially expressed miRNAs according the data of GSE65626 microarray were drawn (Fig. 3a), and it was suggested that *Homo sapiens* (has)-miR-138-2-3p was poorly expressed in glioma tissues. A total of 230 potential miRNAs that may sponged with NCK1-AS1 were predicted via RNA22 with *p* < 0.05 as the selection criterion. Meanwhile, 27 miRNAs with low expression in the microarray GSE65626 were found, among which 3 miRNAs were found as the down-stream miRNAs of NCK1-AS1: hsa-miR-487a-5p, hsa-miR-184 and hsa-miR-138-2-3p (Fig. 3b). The binding sites between NCK1-AS1 and has-miR-138-2-3p predicted on RNA22 are shown in Fig. 3c, indicating NCK1-AS1 might sponge miR-138-2-3p. Moreover, the target genes of miR-138-2-3p were acquired in several databases (RNA22, miRDB, miRDIP, miRWalk and DIANA), and then a Venn diagram was produced (Fig. 3d) with 36 intersections found. The potential intersect genes were further compared with the highly expressed genes in the glioma microarrays GSE50161 and GSE35493, after which 2 intersections TRIM24 and GLISE were found (Fig. 3e). As aforementioned, TRIM24 was noted as an oncogene, but its role in glioma remains unknown. High expression of TRIM24 in microarrays GSE50161 and GSE35493 are shown in Fig. 3f and Fig. 3g. TRIM24 is also highly expressed in the TCGA-GBM DataBase (Fig. 3h). The biding site between miR-138-2-3p and TRIM24 predicted on RNA22 are shown in Fig. 3i. As mentioned above, TRIM24 has been documented to regulate the Wnt/β-catenin signaling pathway. Herein, we speculated that NCK1-AS1 might regulate TRIM24 expression via sponging miR-138-2-3p and further regulate the Wnt/β-catenin pathway in glioma.

The expression of miR-138-2-3p, TRIM24 and β-catenin in glioma and normal brain tissues was measured using RT-qPCR and western blot analysis. It was shown that miR-138-2-3p was lowly expressed, while the mRNA and protein expression of TRIM24 was elevated in glioma tissues (all *p* < 0.05) (Fig. 3j-k). The putative binding sites between NCK1-AS1 and miR-138-2-3p and between miR-138-2-3p and TRIM24 are shown in Fig. 3c and Fig. 3h. The dual luciferase reporter gene assay results suggested that NCK1-AS1 could directly bind to miR-138-2-3p, and miR-138-2-3p could bind to TRIM24 (all *p* < 0.05) (Fig. 3l-m).

### NCK1-AS1 acts as a ceRNA for miR-138-2-3p in glioma cancer

**Fig. 4:**
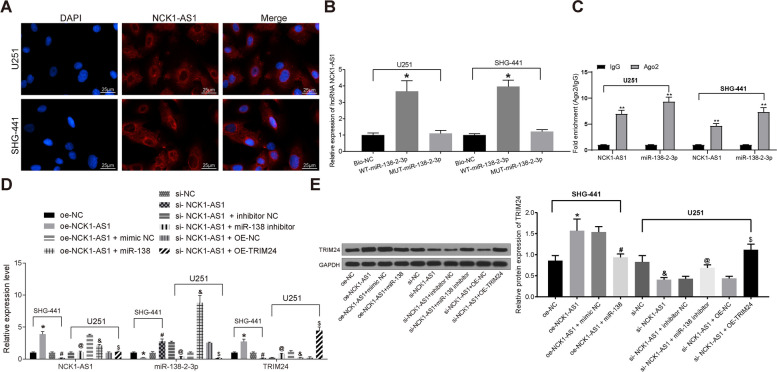
NCK1-AS1 acts as a ceRNA in glioma cancer through regulation of miR-138-2-3p. **a**, sublocalization of NCK1-AS1 in U251 cells detected using FISH; **b** binding of NCK1-AS1 and miR-138-2-3p identified using RNA pull-down assay, * compared to the Bio-NC group, *p* < 0.05; **c** reaction between NCK1-AS1 and AGO2 identified by RIP assay, * compared to the IgG group, p < 0.05; **d-e**, mRNA (**d**) and protein (**e**) expression of NCK1-AS1, miR-138-2-3p and TRIM24 in cells following NCK1-AS1 and miR-138-2-3p interventions measured using RT-qPCR and western blot analysis, respectively, * compared to the oe-NC group, *p* < 0.05; ^#^ compared to the sh-NC group, *p* < 0.05; ^&^ compared to the mimic NC group, *p* < 0.05; ^@^ compared to the inhibitor NC group, *p* < 0.05. Measurement data were measured using mean ± SD; differences among multiple groups were analyzed using one-way ANOVA; Repetition = 3

We further detected the sub-cellular localization of NCK1-AS1 in U251 and SHG-441cells using FISH. As shown in Fig. 4a, in which the blue parts indicate nuclei while the red parts indicate NCK1-AS1, it was shown that NCK1-AS1 is mainly sub-localized in cytoplasm, indicating NCK1-AS1 might exert function through the ceRNA network. Following the findings above, RNA pull-down and RIP assays were further performed to identify the interactions among NCK1-AS1, miR-138-2-3p and TRIM24. The RNA pull-down assay found that compared to the MUT-miR-138-2-3p and the Bio-NC groups, the binding with NCK1-AS1 increased in the WT-miR-138-2-3p group (Fig. 4b). Meanwhile, the RIP assay results suggested that NCK1-AS1 could form compound with anti-AGO2 in U251 and SHG-441 cells (Fig. 4c). Since AGO2 is capable of forming compound with microRNAs, the fact that NCK1-AS1 combined with the AGO2-miR-1382-3p compound further identified the binding relationship between NCK1-AS1 and miR-138-2-3p.

Next, U251 cells with silenced NCK1-AS1 were further transfected with miR-138-2-3p inhibitor, while SHG-441 cells with over-expressed NCK1-AS1 were transfected with miR-138-2-3p mimic. RT-qPCR found that silencing of NCK1-AS1 increased miR-138-2-3p expression in U251 cells, leading to decreased TRIM24 expression. Then, miR-138-2-3p inhibitor transfection partly recovered the TRIM24 expression. In SHG-441 cells, overexpression of NCK1-AS1 led to decreased miR-138-2-3p expression and increased TRIM24 expression, but miR-138-2-3p mimic transfection significantly decreased TRIM24 expression (Figure 4d,e).

### Up-regulation of miR-138-2-3p inhibits invasion and migration but promotes apoptosis of U251 cells via down-regulating TRIM24

**Fig. 5:**
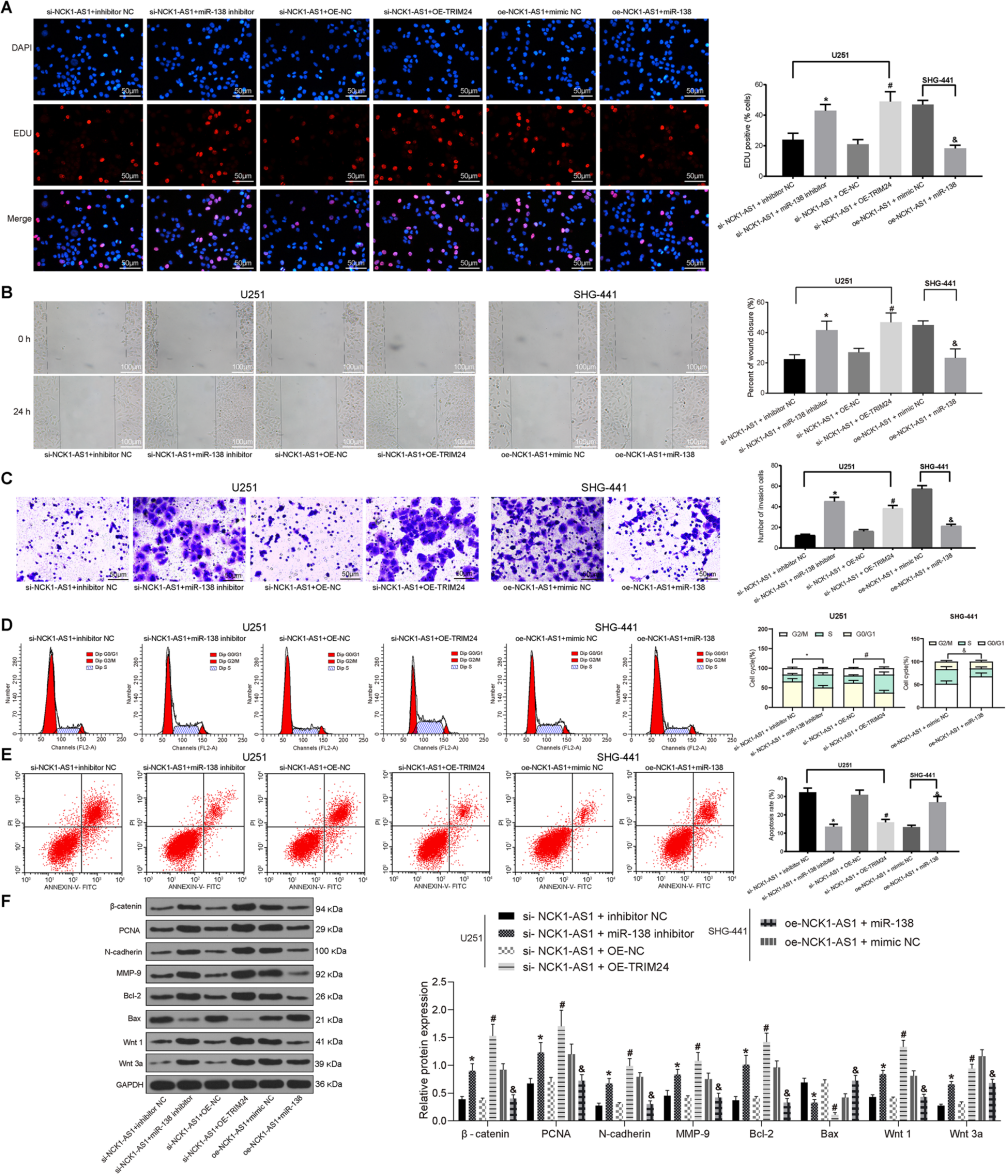
Up-regulation of miR-138-2-3p inhibits invasion and migration but promotes apoptosis of U251 cells via down-regulating TRIM24. **a** proliferation of U251 cells detected using EdU assay; **b** migration of U251 cells detected using the scratch test and observed under the microscope (× 100); **c** number of invaded cells in the Transwell assay observed under the microscope (× 100); **d-e** cell cycle (**d**) and the apoptosis rate (**e**) of U251 cells measured using flow cytometry; **f**, protein levels of Wnt/β-catenin pathway related factor (Wnt1, Wnt3a and β-catenin), proliferation-related factor (PCDA), invasion and migration related factors (N-cadherin and MMP), and apoptosis-related factors (Bcl-2 and Bax) in U251 cells were determined using western blot analysis. * compared to the mimic-NC group, *p* < 0.05; ^#^ compared to the inhibitor-NC group, *p* < 0.05; ^&^ compared to the miR-138-2-3p mimic + oe-NC group, *p* < 0.05. Measurement data were measured using mean ± SD; differences among multiple groups were analyzed using one-way ANOVA; Repetition = 3

Following the above findings, we further identified that inhibition of miR-138-2-3p or over-expression of TRIM24 aggregated the malignant behaviors of U251 cells with silenced NCK1-AS1, including increased cell proliferation, invasion and migration while reduced cell cycle arrest and cell apoptosis. Consistently, up-regulation of miR-138-2-3p inhibited the above malignant behaviors in SHG-441 cells with over-expressed NCK1-AS1 (Fig. 5a-e).

Meanwhile, western blot analysis was applied to measure the protein levels of Wnt/β-catenin pathway-related factor (Wnt1, Wnt3a and β-catenin), proliferation-related factor (PCDA), invasion and migration-related factors (N-cadherin and MMP), and apoptosis-related factors (Bcl-2 and Bax) in cells. The results told silencing of miR-138-2-3p or over-expression of TRIM24 increased the protein levels of Wnt1, Wnt3a, β-catenin, PCNA, N-cadherin, MMP-9 and Bcl-2 while inhibited the Bax expression. But over-expression of miR-138-2-3p in SHG-441 cells led to opposite trends (all *p* < 0.05) (Fig. 5f).

### Silencing of NCK1-AS1 promotes the stemness of glioma cells

**Fig. 6:**
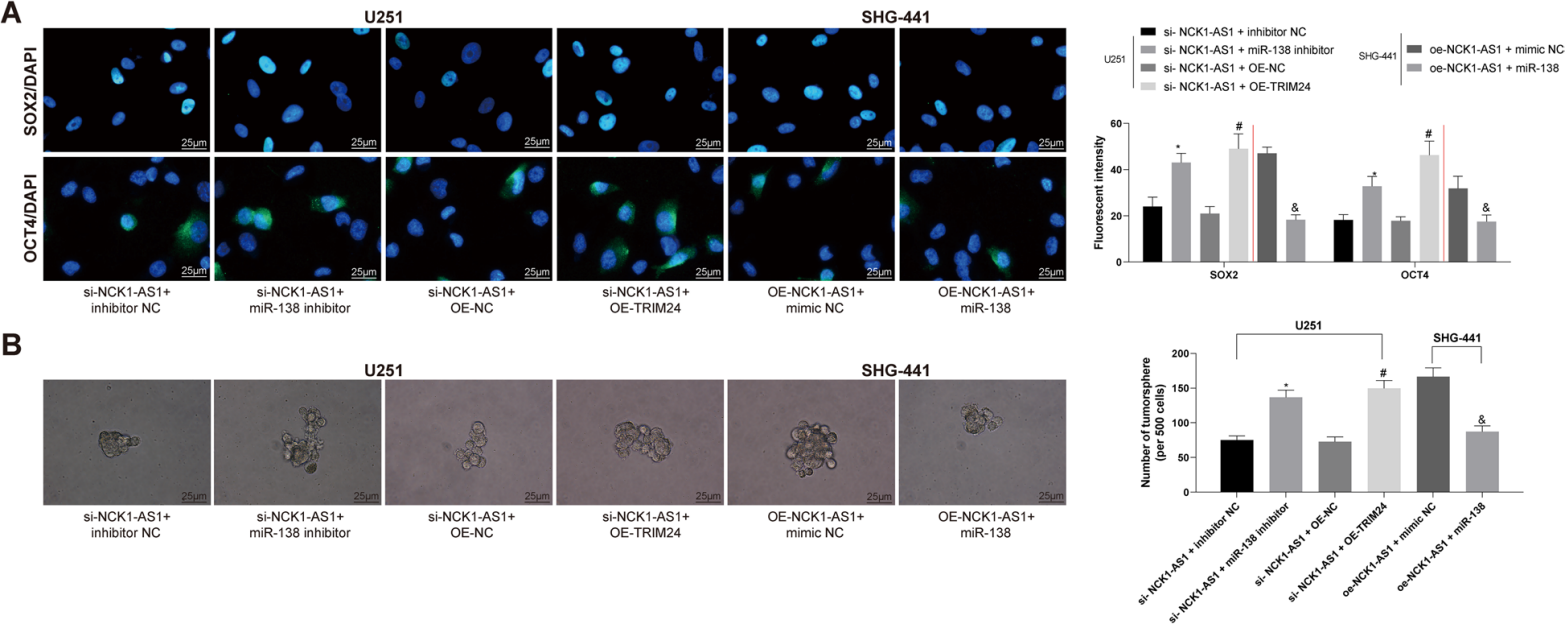
Silencing of NCK1-AS1 promotes the stemness of glioma cells. **a** expression of CSC biomarkers SOX2 and OCT4 determined by immunofluorescence staining; **b** proliferation of U251 and SKH-441 cells measured by tumor sphere formation assay. * compared to the sh-NC group, *p* < 0.05; ^#^ compared to the oe-NC group, *p* < 0.05; ^&^ compared to the oe-NCK1-AS1 + mimic NC group, *p* < 0.05. Measurement data were measured using mean ± SD; differences among multiple groups were analyzed using one-way ANOVA; Repetition = 3

As one of the most malignant tumors, glioma is featured with strong cancer stem cell (CSC) characteristics. Here we determined the CSC-related biomarkers SOX2 and OCT4 in U251 and SHG-441 cells. The results showed that silencing of NCK1-AS1 inhibited the expression of SOX2 and OCT4 in cells, while the further silencing of miR-138-2-3p partly recovered the SOX2 and OCT4 expression. The reverse trends were found in SHG-441 cells with over-expressed NCK1-AS1 and further over-expressed miR-138-2-3p (all *p* < 0.05) (Fig. 6a). Moreover, the similar results were produced in tumor sphere formation assay (all *p* < 0.05) (Fig. 6b).

### Silencing of NCK1-AS1 or up-regulation of miR-138-2-3p inhibits tumor formation in nude mice

**Fig. 7:**
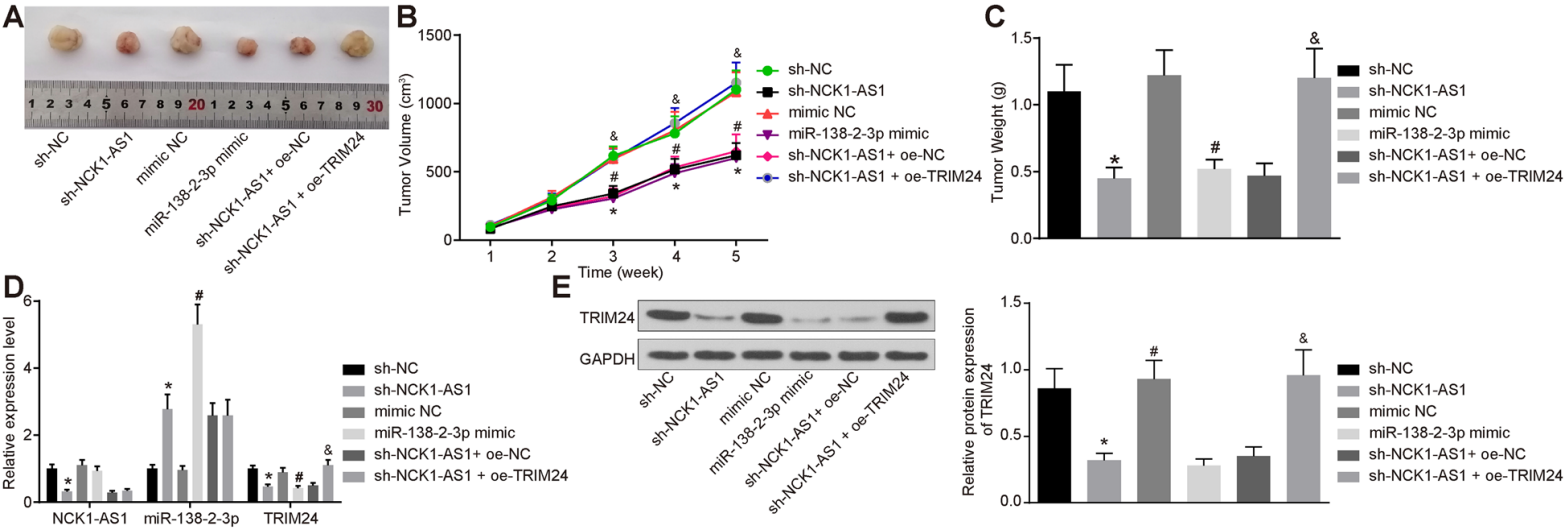
Silencing of NCK1-AS1 or over-expression of miR-138-2-3p inhibits tumor formation in nude mice. **a** morphology of tumor in each group; **b** the volume of tumors in each group; **c** the weight of tumors in each group; **d** expression of NCK1-AS1 and miR-138-2-3p and mRNA expression of TRIM24 in each group of tumor measured using RT-qPCR; **e**, protein level of TRIM24 in each group of tumor evaluated using western blot analysis. * compared to the sh-NC group, *p* < 0.05; ^#^ compared to the mimic-NC group, *p* < 0.05; ^&^ compared to the sh-NCK1-AS1+ oe-NC group, *p* < 0.05. Measurement data were measured using mean ± SD; differences among multiple groups were analyzed using one-way ANOVA; *N* = 5

Nude mice were implanted with cells with stable transfections to further investigate the effect of NCK1-AS1 on tumor formation in vivo. The tumor volume and weight were detected and recorded (Fig. 7a-c). It was found that silencing of NCK1-AS1 or up-regulation of miR-138-2-3p led to increased TRIM24 expression and decreased tumor formation speed in mice, while the further over-expression of TRIM24 promoted the tumor formation speed (all *p* < 0.05) (Figure 7 d,e).

## Discussion

It is quite necessary to develop new potential target for glioma treatment since the overall 5-year survival rate of patients with glioma remains lower than 5% even following comprehensive chemotherapy, radiotherapy and surgery [26]. LncRNAs can sponge miRNAs and block these miRNAs from regulating the target mRNAs, thus mediating target genes post transcriptionally [27]. In light of this theory and several recently identified ceRNA networks in cancers, the study figured out a novel NCK1-AS1/miR-138-2-3p/TRIM24 ceNRA network in glioma.

The evidence concerning the correlation between abnormal lncRNA expression with cancers has been growing [28, 29]. Initially, data on glioma microarrays suggested that NCK1-AS1 is aberrantly highly expressed in glioma, which was further identified by RT-qPCR. As for the other screened out lncRNAs, lncRNA CRNDE, for example, has already been noted to promote glioma cell growth [30], while LINC00998, has hardly been investigated in human cancers. NCK1-AS1 is a relatively newly recognized lncRNA with its oncogenic role in many several human malignancies [19, 31, 32] reported, thus, was selected as the current study subject. Then, we found that silencing of NCK1-AS1 led to decreased proliferation, invasion and migration abilities while increased cell cycle arrest and apoptosis of glioma cells. Moreover, silencing of NCK1-AS1 was also found to inhibit the expression of CSC biomarkers SOX2 and OCT4 in our study. As CSCs are a class of self-renewal cells with strong tumorigenic potency and are more resistant to conventional therapies than other cancer cells [33]. This finding further evidenced the promoting role of NCK1-AS1 in the malignant behaviors of glioma cells. The same trends were reproduced in in vivo experiments, which showed that silencing of NCK1-AS1 inhibited tumor formation and growth in nude mice.

The findings above triggered us to identify the downstream mechanisms involved in the events. As above mentioned, lncRNAs might exert functions through the crosstalk with miRNAs and mRNAs [14, 15]. Therefore, we analyzed the potential target miRNAs of NCK1-AS1 and the following genes via integrated online bioinformation system, glioma microarrays, dual luciferase reporter gene, RNA pull-down and RIP assays, after which we identified the interactions among NCK1-AS1, miR-138-2-3p and TRIM24. Importantly, we noticed that up-regulation of miR-138-2-3p decreased proliferation, invasion and migration while promoted apoptosis of SHG-441 cells with over-expressed NCK1-AS1, showing as decreased levels of PCNA, N-cadherin, MMP-9 and Bcl-2 while increased level of Bax. But artificial over-expression of TRIM24 led to opposite trends in U251 cells with silenced NCK1-AS1. PCNA is an important replication accessory factor that supports DNA replication, repair and recombination, and cell cycle regulation [34, 35]. Abnormal expression of the N-cadherin is a crucial biomarker of epithelial-to-mesenchymal transition in many cancer types, promoting the aggressiveness of tumors [36]. As one of the most studied MMPs, MMP-9 is well-known for the key roles in invasion of cancer cells and metastasis of tumors [37]. Bcl-2 is an anti-apoptotic protein that mediates apoptosis by regulating the permeability of the mitochondrial membrane, while Bax can break the outer mitochondrial membrane thus promoting apoptosis [38]. In addition, hsa-miR-487a-5p and hsa-miR-184 were also identified as NCK1-AS1 targets after integrated analysis. However, the role of miR-184 in glioma has been largely studied [39, 40], while the role of miR-487a-5p in cancer development has been little concerned. miR-138-2-3p is a rarely mentioned miRNA, though, some same trends were found in a previous study [41], which suggested that over-expression of miR-138-2-3p decreased proliferation and invasion but promoted apoptosis of human laryngeal CSCs following radiotherapy. Meanwhile, lowly-expressed miR-138-2-3p has been found in drug-resistant non-small cell lung cancer patients [42]. As for TRIM24, it is a member of the tripartite motif (TRIM) family [20], and the oncogenic role of TRIM24 in carcinogenesis has been demonstrated in several cancer types [43–45]. Moreover, our study identified that inhibited miR-138-2-3p or promoted TRIM24 stimulated Wnt/β-catenin activation in glioma cells, which was in line with the previous studies [21, 41]. Wnt/β-catenin controls diverse cellular processes and drives cancer progression [46], with its activation revealed to promotes tumorigenesis of multiple cancers [47, 48], including glioma [23]. Taken the above discussion together, in can be inferred that NCK1-AS1 could promote the malignant behaviors of glioma cells and tumor metastasis through the crosstalk with miR-138-2-3p and TRIM24, and the following activation of the Wnt/β-catenin pathway.

By the way, GLIS2, the other intersected target gene of miR-138-2-3p, is a member of GLI-similar zinc finger protein family and is closely linked with acute myeloid leukaemia, has been also noted to correlated with tumor progression [49, 50]. We would like to investigate if GLIS2 plays roles in glioma progression in our future experiments.

## Conclusions

**Fig. 8:**
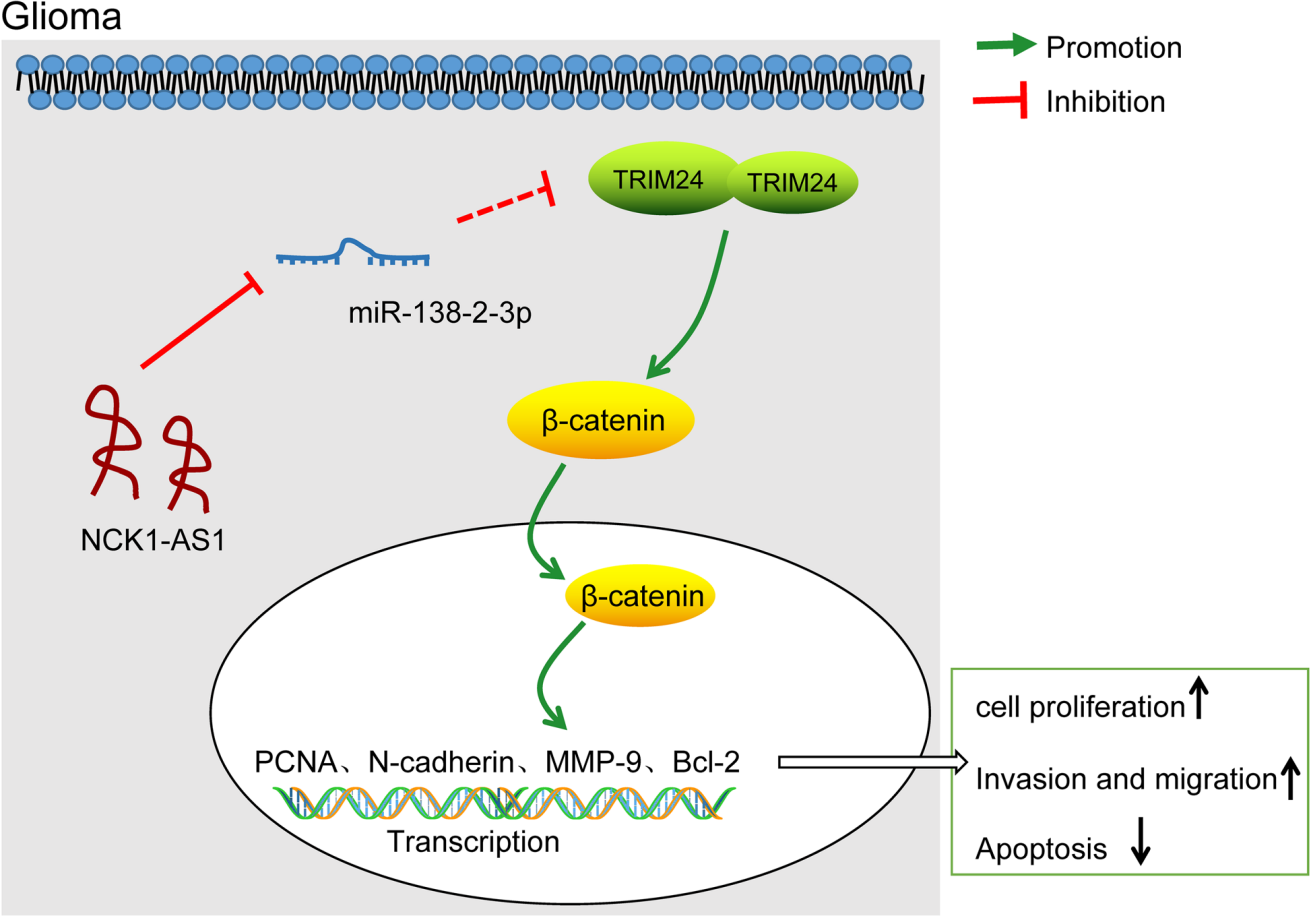
Diagram for the molecular mechanism. In glioma cells, NCK1-AS1 increases TRIM24 expression by sponging miR-138-2-3p. Up-regualtion of TRIM24 promotes the nuclear translocation of β-catenin, leading to increased expression of PCNA, N-cadherin, MMP-P and Bcl-2, resulting in increased cell proliferation, invasion and migration while decreased apoptosis

To sum up, this study identified a novel ceRNA network in glioma that NCK1-AS1 up-regulates TRIM24 expression through sponging miR-138-2-3p. Silencing of NCK1-AS1 might inhibit the proliferation, invasion, migration and resistance to death of glioma cells, and inhibit the metastasis of tumor in vivo through down-regulating TRIM24 and the following Wnt/β-catenin inactivation (Fig. 8). These findings might provide novel insights in glioma treatment, and we hope more studies in this field would be carried out to validate our findings, and to identify more potential mechanisms for better understanding of glioma.

### Supplementary information

**Additional file 1 Supplementary Table 1.** Sequences of transfecting vectors.

## Data Availability

All the data generated or analyzed during this study are included in this published article.

## Abbreviations

AGO2: Argonaute 2
ANOVA: Analysis of variance
Bax: Bcl-2-associated X
Bcl-2: B-cell lymphoma-2
BSA: Bovine serum albumin
DMEM: Dulbecco’s modified eagle’s medium
ECM: Extracellular matrix
EDU: 5-ethynyl-2′-deoxyuridine
FITC: Fluorescein isothiocyanate
FBS: Fetal bovine serum
FISH: Fluorescence in situ hybridization;
GAPDH: Glyceraldehyde-3-phosphate dehydrogenase
HRP: Horseradish peroxidase
has: *Homo sapiens*
IgG: Immunoglobulin G
mean ± SD: mean ± standard derivation
MMP-9: Matrix metalloproteinase-9
MUT: Mutant type
NC: Negative control.
PBS: Phosphate buffer saline
PCNA: Proliferating cell nuclear antigen
PI: Propidium iodide
RIP: RNA immunoprecipitation
RIPA: Radio-immunoprecipitation assay
RT-qPCR: reverse transcription quantitative polymerase chain reaction
SDS-PAGE: Sodium dodecyl sulfate-polyacrylamide gel electrophoresis
TRIM24: Tripartite motif containing 24
WT: Wild type
