# Correction: Long non-coding RNA NCK1-AS1 promotes the tumorigenesis of glioma through sponging microRNA-138-2-3p and activating the TRIM24/Wnt/β-catenin axis

**DOI:** 10.1186/s13046-023-02819-6

**Published:** 2023-09-21

**Authors:** Lifa Huang, Xu Li, Hui Ye, Yajun Liu, Xiaolong Liang, Chao Yang, Lin Hua, Zhaoxian Yan, Xin Zhang

**Affiliations:** 1 Department of Neurosurgery, Zhejiang Provincial Hospital of Traditional Chinese Medicine/The First Affiliated Hospital of Zhejiang Chinese Medical University, No. 54, Youdian Road, Shangcheng District, Hangzhou, Zhejiang 310006 People’s Republic of China; 2 The First Clinical Medical College, Zhejiang Chinese Medical University, Hangzhou, Zhejiang 310053 People’s Republic of China

**Correction:** ***J Exp Clin Cancer Res*** **39, 63 (2020)**

10.1186/s13046-020-01567-1

Following publication of the original article [1], the authors would like to correct the Funding section from ‘**This study was supported by the National Natural Science Foundation of China (81873186).**’ to ‘**None.**’

The correction does not affect the overall result or conclusion of the article. The original article has been corrected.
