# Supplementary material for: Long non-coding RNA NCK1-AS1 promotes the tumorigenesis of glioma through sponging microRNA-138-2-3p and activating the TRIM24/Wnt/β-catenin axis
Source: J Exp Clin Cancer Res. 2020 Apr 15;39:63. doi: 10.1186/s13046-020-01567-1 (PMC7158134; doi:10.1186/s13046-020-01567-1)
Supplement: Supplementary file 1 — Additional file 1 Supplementary Table 1. Sequences of transfecting vectors. [file 13046_2020_1567_MOESM1_ESM.docx]

**Supplementary Table 1** Sequences of transfecting vectors

| Vector/Gene | Sequence (5'-3') |
| --- | --- |
| sh-NC | UUCUCCGAACGUGUCACGUTT |
| sh-NCK1-AS1 | GAAUGUCAUCCCAGCCGAAT |
| mimic NC | GGCAGGUAACGUGCGUUUCGA |
| miR-138-2-3p | AGCUGGUGUUGUGAAUCAGGCCG |
| oe-NC | UUCUCCGAACGUGUCACGUTT |
| oe-TRIM24 | ACTCGGGACATACCTGCTCT |
| oe-NCK1-AS1 | GATCCGAATGTCATCCCAGCCGAATTCAAGAGATTCGGCTGGGATGACATTCTTTTTTG |

Note: sh, short hairpin; NC, negative control; oe, over-expression; TRIM24, tripartite motif-containing 24.
